# SP7 Inhibits Osteoblast Differentiation at a Late Stage in Mice

**DOI:** 10.1371/journal.pone.0032364

**Published:** 2012-03-02

**Authors:** Carolina A. Yoshida, Hisato Komori, Zenjiro Maruyama, Toshihiro Miyazaki, Keishi Kawasaki, Tatsuya Furuichi, Ryo Fukuyama, Masako Mori, Kei Yamana, Kouhei Nakamura, Wenguang Liu, Satoru Toyosawa, Takeshi Moriishi, Hiroshi Kawaguchi, Kenji Takada, Toshihisa Komori

**Affiliations:** 1 Department of Cell Biology, Nagasaki University Graduate School of Biomedical Sciences, Nagasaki, Japan; 2 Department of Orthopaedic Surgery, Faculty of Medicine, The University of Tokyo, Tokyo, Japan; 3 Department of Orthodontics and Dentofacial Orthopedics, Graduate School of Dentistry, Osaka University, Suita, Japan; 4 Laboratory of Pharmacology, Faculty of Pharmaceutical Sciences, Hiroshima International University, Kure, Japan; 5 Teijin Institute for Biomedical Research, Teijin, Tokyo, Japan; 6 Department of Periodontology, Nagasaki University Graduate School of Biomedical Sciences, Nagasaki, Japan; 7 Department of Oral Pathology, Graduate School of Dentistry, Osaka University, Suita, Japan; University of Massachusetts Medical, United States of America

## Abstract

RUNX2 and SP7 are essential transcription factors for osteoblast differentiation at an early stage. Although RUNX2 inhibits osteoblast differentiation at a late stage, the function of SP7 at the late stage of osteoblast differentiation is not fully elucidated. Thus, we pursued the function of SP7 in osteoblast differentiation. RUNX2 induced *Sp7* expression in *Runx2* ^−/−^ calvarial cells. Adenoviral transfer of sh-*Sp7* into primary osteoblasts reduced the expression of *Alpl*, *Col1a1*, and *Bglap2* and mineralization, whereas that of *Sp7* reduced *Bglap2* expression and mineralization at a late stage of osteoblast differentiation. *Sp7* transgenic mice under the control of 2.3 kb *Col1a1* promoter showed osteopenia and woven-bone like structure in the cortical bone, which was thin and less mineralized, in a dose-dependent manner. Further, the number of processes in the osteoblasts and osteocytes was reduced. Although the osteoblast density was increased, the bone formation was reduced. The frequency of BrdU incorporation was increased in the osteoblastic cells, while the expression of *Col1a1*, *Spp1*, *Ibsp*, and *Bglap2* was reduced. Further, the osteopenia in *Sp7* or *Runx2* transgenic mice was worsened in *Sp7/Runx2* double transgenic mice and the expression of *Col1a1* and *Bglap2* was reduced. The expression of *Sp7* and *Runx2* was not increased in *Runx2* and *Sp7* transgenic mice, respectively. The expression of endogenous *Sp7* was increased in *Sp7* transgenic mice and *Sp7*-transduced cells; the introduction of *Sp7* activated and sh-*Sp7* inhibited *Sp7* promoter; and ChIP assay showed the binding of endogenous SP7 in the proximal region of *Sp7* promoter. These findings suggest that SP7 and RUNX2 inhibit osteoblast differentiation at a late stage in a manner independent of RUNX2 and SP7, respectively, and SP7 positively regulates its own promoter.

## Introduction

After multipotent mesenchymal cells commit to the osteoblastic lineage, preosteoblasts differentiate into osteoblasts, which express bone matrix proteins including COL1A1, COL1A2, SPP1 (osteopontin), IBSP (bone sialoprotein), and BGLAP2 (osteocalcin) [1]. RUNX2, SP7/Osterix, and canonical Wnt signaling are essential for the commitment of mesenchymal cells to the osteoblastic lineage [2], [3]. The osteoblasts then express bone matrix protein genes at different levels depending on the maturity of the cells. Mesenchymal cells and preosteoblasts weakly express *Col1a1* and *Col1a2*, but osteoblasts showed increased levels. Immature osteoblasts express *Spp1* and then *Ibsp*, and mature osteoblasts strongly express *Bglap2* [1]. Mature osteoblasts are embedded into the bone matrix and become osteocytes. Osteocytes, the most abundant cells in mature bone, are extensively interconnected with other osteocytes and osteoblasts through processes [4].

SP7 is a zinc finger-containing protein which belongs to the Sp/KLF (Kruppel like Factor) family of transcription factors [5]. *Sp7*-deficient (*Sp7* ^−/−^) mice are unable to form bone due to an arrest of osteoblast differentiation. Their mesenchymal cells condense in the perichondrial region, where bone normally forms, and express *Runx2* and genes for chondrocyte markers but not bone matrix proteins. Therefore, SP7 is essential for osteoblast differentiation, and *Runx2* ^+^ *Sp7^−^* mesenchymal cells (preosteoblasts) retain the ability to differentiate into chondrocytes [5]. Further, osteoblast-specific deletion of *Sp7* resulted in osteopenia due to the inhibition of osteoblast differentiation in adult mice [6]. However, the function of SP7 after the commitment to the osteoblastic lineage is not fully elucidated.

To pursue this issue, we over-expressed or silenced *Sp7*. Introduction of sh-*Sp7* inhibited osteoblast differentiation from the early stage of osteoblast differentiation, but introduction of *Sp7* inhibited osteoblast differentiation at a late stage in vitro. Osteoblast-specific *Sp7* transgenic mice showed osteopenia due to the inhibition of osteoblast differentiation at a late stage, and SP7 positively regulated its own promoter. These findings suggest that the regulation of *Sp7* expression depending on the stage of osteoblast differentiation is important for bone development and maintenance.

## Materials and Methods

### Ethics statement

Prior to the study, all experiments were reviewed and approved by the Animal Care and Use Committee of Nagasaki University Graduate School of Biomedical Sciences, permit number (0906170767-5).

### Cell culture experiments

Calvaria from *Runx2* ^−/−^ and wild-type embryos at E18.5 were embedded in a type I collagen gel matrix for 10 days and the cells outspreading from the explants were collected using 0.1% collagenase. The cells were then plated on 24-well plates at a density of 5×10^4^/well. At confluence, they were infected with an adenovirus expressing EGFP, *Runx2*, or *Sp7*. In vitro silencing of *Sp7* was obtained by adenoviral delivery of sh-RNA containing the target sequence of ACAAAGAAGCCATACGCT. Primary osteoblasts were isolated from calvaria of wild-type mice at E18.5 by sequential digestion with 0.1% collagenase A and 0.2% dispase. Osteoblastic cells from the second to fifth fraction were pooled, and plated on 24-well plates at a density of 5×10^4^/well. At confluence, the osteoblastic cells were infected with an adenovirus expressing EGFP, *Sp7*, sh-*Sp7*, or *Runx2*. Cell viability evaluation and retrovirus infection in primary osteoblasts were performed as described in the Supporting Information files (Procedures S1 and S2). To induce osteogenic differentiation the cells were cultured in alfa-MEM (Sigma) containing 10%FBS (JRH), 10 mM β-glycerophosphate, and 50 μg/ml ascorbic acid. The staining for mineralization was performed 10 days after the infection as described previously [7].

### Generation of *Sp7* transgenic mice and *Runx2/Sp7* double transgenic mice

To generate transgenic mice that overexpress *Sp7* in osteoblasts, *Sp7* cDNA was inserted into the mammalian expression vector pNASSβ (CLONTECH Laboratories Inc., Otsu, Japan) by replacing the β-galactosidase gene at the Not I sites, and the 2.3-kb osteoblast-specific promoter region of mouse *Col1a1* [8] was inserted into the pNASSβ at the XhoI site. To generate *Runx2/Sp7* double transgenic mice, tg2 mice were mated with transgenic mice that overexpress *Runx2* under the control of the 2.3-kb mouse *Col1a1* promoter [9]. The urinary excretion of deoxypyridinoline was measured using an ELISA kit (Metra Biosystems). Results were expressed as deoxypyridinoline/creatinine (nmol/mmolCr).

### Histological analyses

For histological analyses, mice were fixed in 4% paraformaldehyde/0.1 M phosphate buffer, and the long bones were decalcified in 0.5 M EDTA/10% glycerol buffer (pH7.5) and embedded in paraffin. Sections (7 μm thick) were stained with hematoxylin and eosin (H–E) or subjected to immunohistochemistry using anti-SP7 antibody [10], Bone canalicular staining (silver impregnation staining) was performed according to the method previously described [11]. For bone histomorphometric analyses, the mice received two injections of calcein at 6 days and 2 days before sacrifice, and longitudinal sections from the distal parts of femurs were analyzed as reported [12]. The BrdU incorporation experiment was performed as detailed previously [13].

### Real-time RT-PCR

Muscle, connective tissue, and periosteum were removed from femurs and tibiae, and the bones were cut at the metaphyses. After hematopoietic cells in the diaphyses of femurs and tibiae were flushed out with PBS, osteoblasts were removed using a micro-intertooth brush (Kobayashi Pharmaceutical Co. Ltd., Osaka, Japan). The remaining bone was used as a source of osteocyte-enriched cells. Nearly complete removal of osteoblasts from the endosteum by the micro-intertooth brush was confirmed using a scanning electron microscope (Miniscope TM-1000; Hitachi). Real-time RT-PCR was performed as described previously [13] using the following primers: transgene, 5′-CCAGAAAGTTAACTGGCCTGT-3′/5′-ATTCCGCAGCTTTTAGAGCAG-3′; *Col1a1*, 5′-CCCAAGGAAAAGAAGCACGTC-3′/5′-ACATTAGGCGCAGGAAGGTCA-3′; *Alpl*, 5′-CGCACGCGATGCAACACCAC-3′/5′- TGCCCACGGACTTCCCAGCA 3′-; *Spp1*, 5′-CTCCAATCGTCCCTACAGTCG-3′/5′-CCAAGCTATCACCTCGGCC-3′; *Ibsp*, 5′-TGGCGACACTTACCGAGCTT-3′/5′-CCATGCCCCTTGTAGTAGCTGTA-3′; *Bglap2*, 5′-CCTAGCAGACACCATGAG-3′/5′-TCTGATAGCTCGTCACAAG-3′; endogenous *Sp7*, 5′- CATCTGCCTGACTCCTTGGGAC-3′/5′-GCTGAAAGGTCAGCGTATGGC-3′; *Sp7* (endogenous and exogenous *Sp7*), 5′-AGGCACAAAGAAGCCATAC-3′/5′-AATGAGTGAGGGAAGGGT-3′; *Runx2* (endogenous and exogenous *Runx2*), 5′-CTTCGTCAGCATCCTATCAGTTC-3′/5′-TCAGCGTCAACACCATCATTC-3′; *Dmp1*, 5′-GGCTGTCCTGTGCTCTCCCAG-3′/5′-GGTCACTATTTGCCTGTGCCTC-3′; *Sost*, 5′-CTTCAGGAATGATGCCACAGAGGT-3′/5′-ATCTTTGGCGTCATAGGGATGGTG-3′; *Fgf23*, 5′-ACTTGTCGCAGAAGCATC-3′/5′-GTGGGCGAACAGTGTAGAA-3′; *Mepe*, 5′-CAGTGGCTCCCCAGATCTTC-3′/5′-GCTTTCAGGACCAGACCCAG-3′; *Phex*, 5′-GTGCATCTACCAACCAGATACG-3′/5′-TCTGTTCCCCAAAAGAAAGG-3′; *Tnfsf11*, 5′-CAAGCTCCGAGCTGGTGAAG-3′/5′-CCTGAACTTTGAAAGCCCCA-3′; *Tnfrsf11b*, 5′-AAGAGCAAACCTTCCAGCTGC-3′/5′-CACGCTGCTTTCACAGAGGTC-3′; *Atf4*, 5′-ACCTCATGGGTTCTCCAGC-3′/5′-CCATTCGAAACAGAGCATCGA-3′. CT (number of cycles at which the threshold of detection was reached) values were normalized to that for rodent *Gapdh* (Applied Biosystems) expression.

### X-ray and pQCT analyses

Long bones were dissected from sacrificed mice at 6 weeks of age and exposed to X-rays using a Micro-FX1000 (Fuji Film Inc., Tokyo, Japan). For the pQCT analysis, femurs from 10-week-old mice were fixed with 70% ethanol and measured using an XCT Research SA (Stratec Medizintechnick, Pforzheim, Germany) as described [9].

### Ultrastructural analysis

Mice were fixed with a mixture of 2% paraformaldehyde and 2.5% glutaraldehyde in 0.05 M cacodylate buffer (pH 7.4). After decalcification, the specimens were post-fixed with osmium tetroxide (OsO_4_), dehydrated, and embedded in Epon-Araldite resin. The ultrathin sections were stained with uranyl acetate and lead citrate, and examined under a transmission electron microscope (H-7100, Hitachi, Tokyo, Japan). To observe the three-dimensional ultrastructure of osteocytes, the HCl-collagenase method [14] was applied. Briefly, the fixed specimens were treated with 6N HCl at 60°C for 50 min, rinsed in 0.1 M phosphate buffer (pH6.8), and placed in 1 mg/ml collagenase (Sigma, Type II; 0.1 M phosphate buffer, pH 6.8) at 37°C for 12 hr. Then, they were immersed in 2% tannic acid solution for 30 min, post-fixed with 1% OsO_4_ in 0.05 M cacodylate buffer (pH 7.4) at 4°C for 1 hr, dehydrated, critical point-dried in CO_2_ (HCP-2, Hitachi), coated with gold, and observed under a scanning electron microscope (S-3500N, Hitachi).

### Reporter assays

The 1.8-kb promoter region of the mouse *Sp7* gene was amplified from mouse genomic DNA by PCR using the following primers, 5′-AAGCTTTTCCCACTCTCTAC-3′/5′-CGAGCTGGGGACCGGGTCCC-3′ and cloned into a firefly luciferase (FL) reporter vector PGL4.10Luc2 (Promega). The deletion fragments were also subcloned into PGL4.10Luc2. 293 cells were co-transfected with the *Sp7* promoter construct, the vector expressing *Sp7*, and a promoter-less Renilla Luciferase vector which was derived from a pRL-SV40 vector (Promega) by deletion of the SV40 promoter. ATDC5 cells were transfected with the 1.8-kb *Sp7* promoter construct and the pRL plasmid, and infected with an adenovirus expressing EGFP or sh-*Sp7*. All plasmid transfections were performed using FuGENE 6 (Roche Diagnostics). Luciferase activity was normalized to that of Renilla luciferase.

### Chromatin immunoprecipitation (ChIP) assay

ChIP was performed with a Magna ChIP G Kit (Millipore, Billerica, MA) using wild-type newborn calvarial cells. After immunoprecipitation with anti-SP7 (Abcam, Tokyo, Japan) or anti-IgG (Cell signaling, Tokyo, Japan) antibody and DNA extraction, PCR was performed using the primers, 5′-CCTCAAGCAGAGAGGACGCCA-3′/5′-TCCGGAGTCTTCTCCGCTGG-3′. An aliquot of sonicated DNA before immunoprecipitation was amplified by PCR as a positive control.

### Statistical analysis

Data are represented as the mean±S.D. Means of groups were compared by ANOVA, and the significance of differences was determined by post-hoc testing with Bonferroni's method.

## Results

### RUNX2 induced *Sp7* expression and sh-*Sp7* inhibited osteoblast differentiation, but SP7 inhibited osteoblast differentiation at a late stage *in vitro*

**Figure 1:**
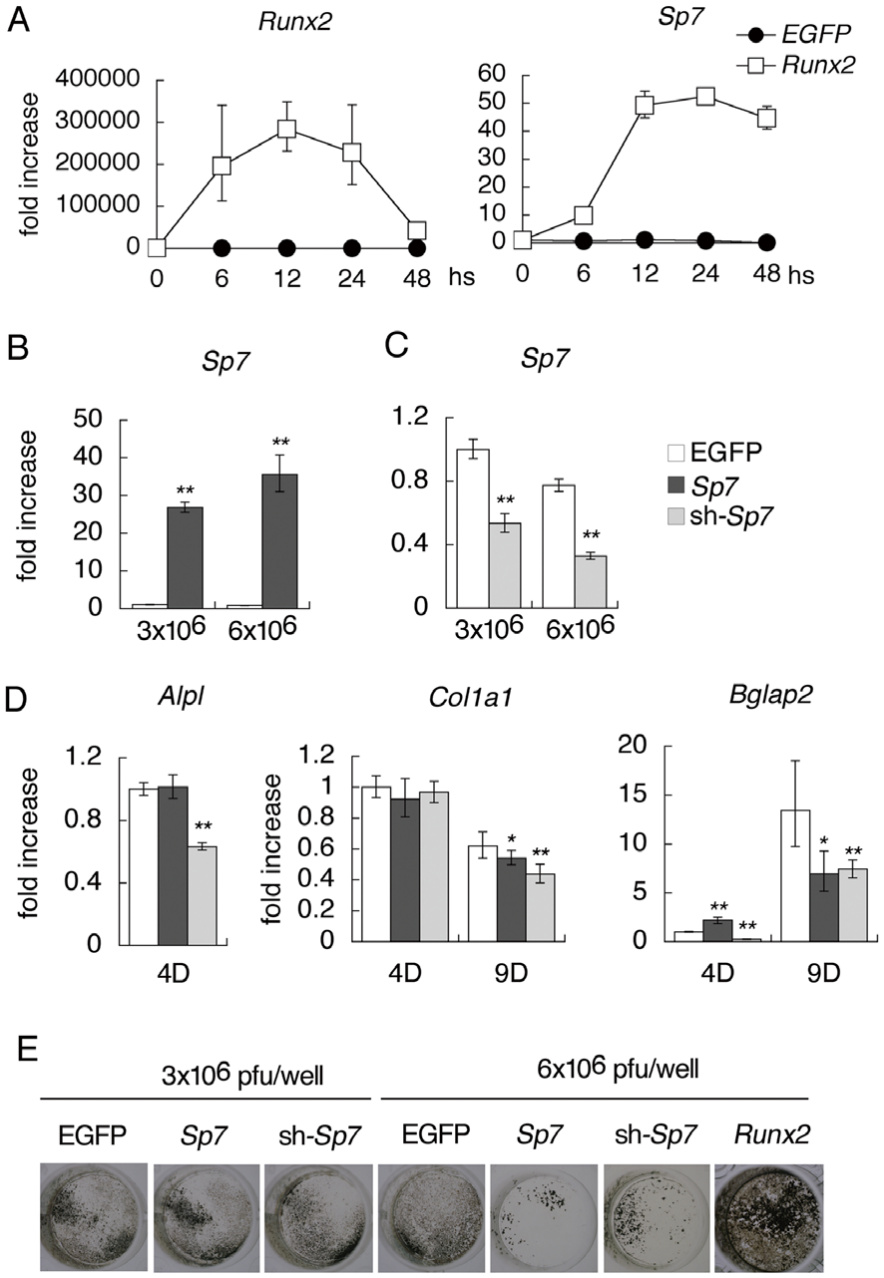
Osteoblast differentiation *in vitro*. (A–D) Real-time RT-PCR analysis. A, Induction of *Sp7* expression by RUNX2 in *Runx2* ^−/−^ calvarial cells. The cells isolated using collagen gel were infected with EGFP or *Runx2*-EGFP expressing adenovirus (6×10^6^ pfu/well). RNA samples were obtained at 6, 12, 24 and 48 hours after infection. Expression levels before infection (0) were defined as 1 and relatives levels are shown. *Runx2* ^−/−^ calvarial cells express an aberrant transcript of *Runx2* at a low level. n = 3. B, *Sp7* expression after the infection of adenovirus carrying EGFP or *Sp7*. C, *Sp7* expression after the infection of adenovirus carrying EGFP or sh-*Sp7*. Adenoviruses were used at 3×10^6^ pfu/well or 6×10^6^ pfu/well as indicated. D, *Alpl*, *Col1a1*, and *Bglap2* expressions were examined at 4 days or 9 days of culture after the infection. Adenoviruses were used at 3×10^6^ pfu/well. The levels in cells, which were infected with EGFP-expressing adenovirus at 3×10^6^ pfu/well, at 4 days of culture were defined as 1, and relative levels are shown. *p<0.05 and **p<0.001 vs. EGFP. n = 4. (E) Von Kossa staining in primary osteoblasts infected with an adenovirus carrying EGFP, *Sp7*, sh-*Sp7*, or *Runx2*. Three independent experiments were performed and representative data are shown in A–E.

As both RUNX2 and SP7 are required for osteoblast differentiation and *Runx2* is expressed in the mesenchymal condensation in *Sp7* ^−/−^ mice [5], we first examined whether RUNX2 is able to induce *Sp7* expression in osteoprogenitors. The infection of *Runx2*-expressing adenovirus into *Runx2* ^−/−^ calvarial cells upregulated *Sp7* expression 10 times after 6 hours and 50 times after 12–48 hours compared with that before infection (Fig. 1A). Next, we examined the ability of SP7 in osteoblastogenesis in vitro. On real-time RT-PCR analysis, the introduction of *Sp7* shRNA (sh-*Sp7*) into primary osteoblasts reduced alkaline phosphatase (*Alpl*) expression at day 4, *Col1a1* expression mildly at day 9, and *Bglap2* expression at days 4 and 9, while the introduction of *Sp7* had no effect on *Alpl* expression, mildly reduced *Col1a1* expression at day 9, and induced *Bglap2* at day 4 but inhibited it at day 9 (Fig. 1B–D). Although the introduction of *Runx2* into primary osteoblasts apparently induced mineralization, both *Sp7* and sh-*Sp7* reduced mineralization at the higher dose (Fig. 1E). The cells were viable at a similar level after the infection of the adenovirus expressing either EGFP, *Sp7*, or sh-*Sp7* at the higher dose (Fig. S1A). Further, retroviral introduction of *Sp7* also inhibited mineralization (Fig. S1B–E). These results suggest that SP7 is required for osteoblast differentiation, but that SP7 is not sufficient to induce osteoblast differentiation and overexpression of *Sp7* suppresses osteoblast differentiation at a late stage in vitro.

### Generation of *Sp7* transgenic mice

**Figure 2:**
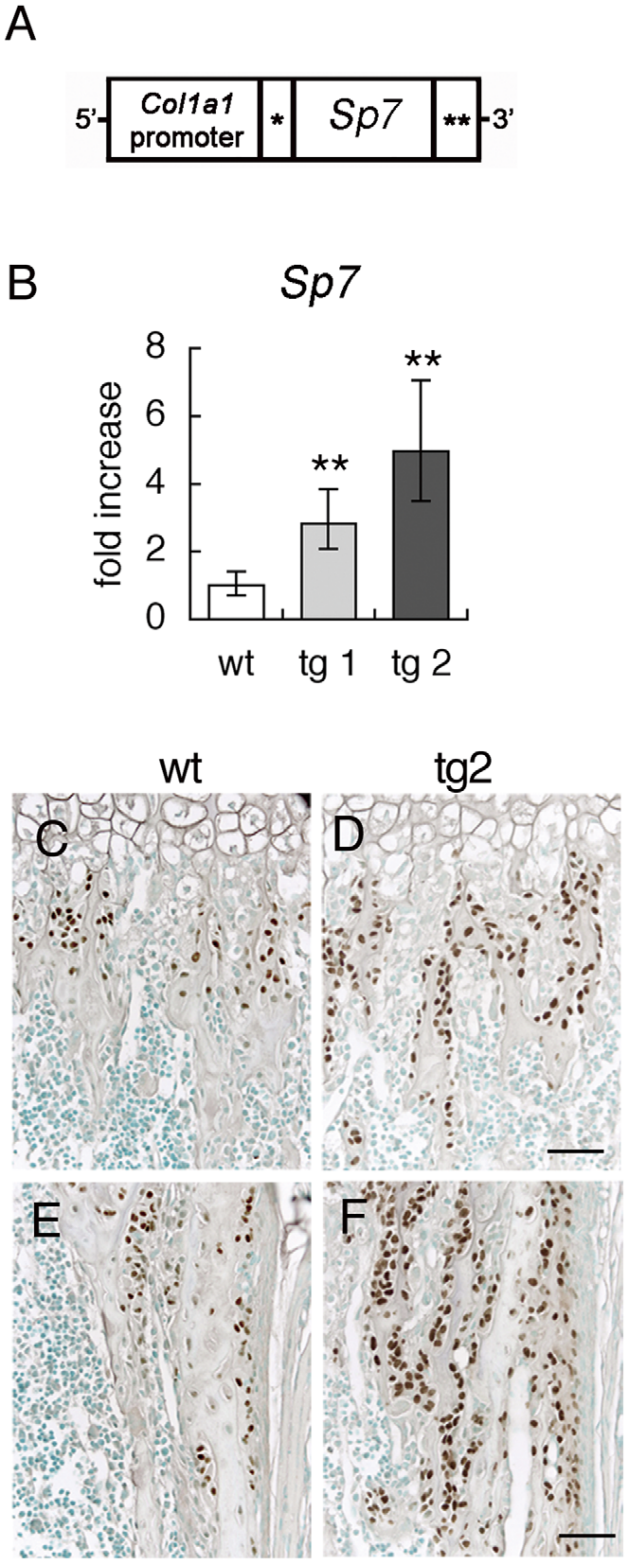
Generation of *Sp7* transgenic mice. (A) Diagram of the DNA construct used to generate *Sp7* transgenic mice. *Intron from SV40 containing splice donor and acceptor sites, **polyadenylation signal from SV40. (B) *Sp7* expression in long bones of wild-type mice and two independent *Sp7* transgenic lines (tg1 and tg2) at 4 weeks of age. *Sp7* expression was examined by real-time RT-PCR. The level in wild-type mice was set at 1, and relative levels are shown. **P<0.01 vs. wild-type mice. n = 4–10. (C–F) Immunohistochemistry using anti-SP7 antibody. Trabecular bone (C, D) and cortical bone (E, F) in the femur of a wild-type mouse (C, E) and tg2 mouse (D, F) at 1 week of age are shown. Bars: 50 μm.

We generated *Sp7* transgenic mice with the mouse 2.3-kb *Col1a1* promoter, which specifically directs transgene expression to immature and mature osteoblasts (Fig. 2A). We established two lines of *Sp7* transgenic mice, tg1 with low transgene expression and tg2 with high transgene expression (Fig. 2B). SP7 expression was examined using anti-SP7 antibody (Fig. 2C–F). The transgene expression was detected in most osteoblasts and young osteocytes but not in osteoclasts and chondrocytes in *Sp7* transgenic mice. The staining was not detected in other tissues except teeth, in which odontoblasts were strongly stained (data not shown). These results confirmed that the transgene was specifically expressed in osteoblasts, early osteocytes, and odontoblasts by the mouse 2.3-kb *Col1a1* promoter as described previously [8], [9].

### *Sp7* transgenic mice show osteopenia

**Figure 3:**
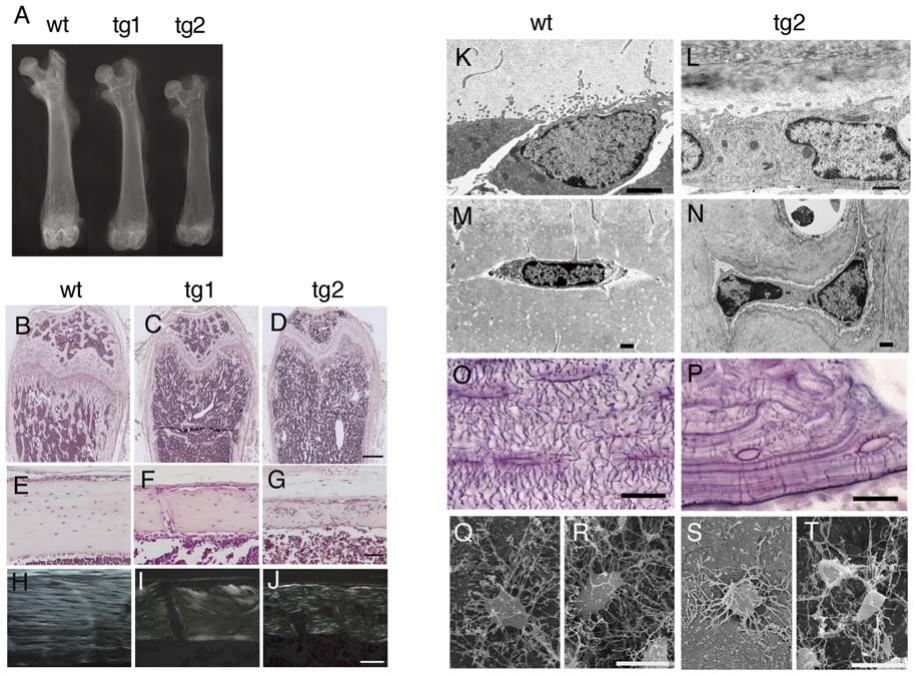
X-ray and histological analyses of *Sp7* transgenic mice. (A) X-ray analysis of the femurs of a wild-type (wt), tg1, and tg2 mouse at 6 weeks of age. (B–G) Histological appearance of bone in transgenic mice. Longitudinal sections through the distal part (B–D) and diaphysis (E–G) of femurs in a wild-type (B, E), tg1 (C, F), and tg2 (D, G) mouse at 6 weeks of age. The sections were stained with H–E. (H–J) Polarized microscopic examination of the diaphyses of femurs from wild-type (H), tg1 (I), and tg2 (J) mice at 6 months of age. (K–T) TEM images of osteoblasts (K, L) and osteocytes (M, N), canalicular staining of tibiae (O, P), and SEM images of preosteocytes (Q, S) and young osteocytes (R, T) of wild-type (K, M, O, Q, R) and tg2 (L, N, P, S, T) mice at 10 weeks of age. In Q–T, bone and osteoid in cortical bone were dissolved and preosteocytes and young osteocytes just beneath the osteoblast layer in the endosteum were observed by SEM. Bars: (B–D) 500 μm; (E–J) 50 μm; (K–N), 1 μm; (O–T), 10 μm.

The two transgenic lines showed osteopenia, the severity of which depended on the expression level of the transgene, and fractures in the lower limbs were sometimes observed in tg2 but not observed in tg1 by X-ray analysis (Fig. 3A and data not shown). The osteopenic phenotype was mild at 3 days of age and apparent after 1 week of age, but was not apparent at the newborn stage in tg2 mice, and both the trabecular and cortical bones were decreased with greater severity in tg2 than tg1 until 10 months of age we examined (Fig. 3B–G, Fig. S2, and data not shown). The direction of collagen fibers was uniform and osteocytes were regularly located in a similar direction in the cortical bone of wild-type mice, whereas the collagen fibers were randomly oriented similar to those in woven bone and the osteocytes were irregularly located and randomly oriented in tg1 and tg2 with milder abnormalities in tg1 (Fig. 3H–J).

On transmission electron microscopy (TEM), the osteoblasts in 10-week-old wild-type mice had spread many processes into osteoid, whereas the number of processes on osteoblasts was reduced in *Sp7* transgenic mice, probably showing the reduced activity of osteoblasts (Fig. 3K, L). The mature osteocytes, which reside in the lacunae, were flattened and had abundant processes in wild-type mice, while two osteocytes were frequently observed in one lacuna, they were irregularly shaped, and the number of processes was less in *Sp7* transgenic mice (Fig. 3M, N). In canalicular staining, abundant canaliculi, through which the processes of osteocytes interact with each other, regularly ran among osteocyte lacunae and between osteocyte lacunae and bone surface in wild-type mice (Fig. 3O), whereas the number of canaliculi was reduced and they were irregularly oriented in *Sp7* transgenic mice (Fig. 3P). On scanning electron microscopy (SEM), preosteocytes, which are in the process of becoming embedded into bone, and young osteocytes had abundant processes in wild-type mice (Fig. 3Q, R), while those in *Sp7* transgenic mice had a reduced number of processes (Fig. 3S, T). Thus, overexpression of *Sp7* reduced the number of processes in both osteoblasts and osteocytes.

**Figure 4:**
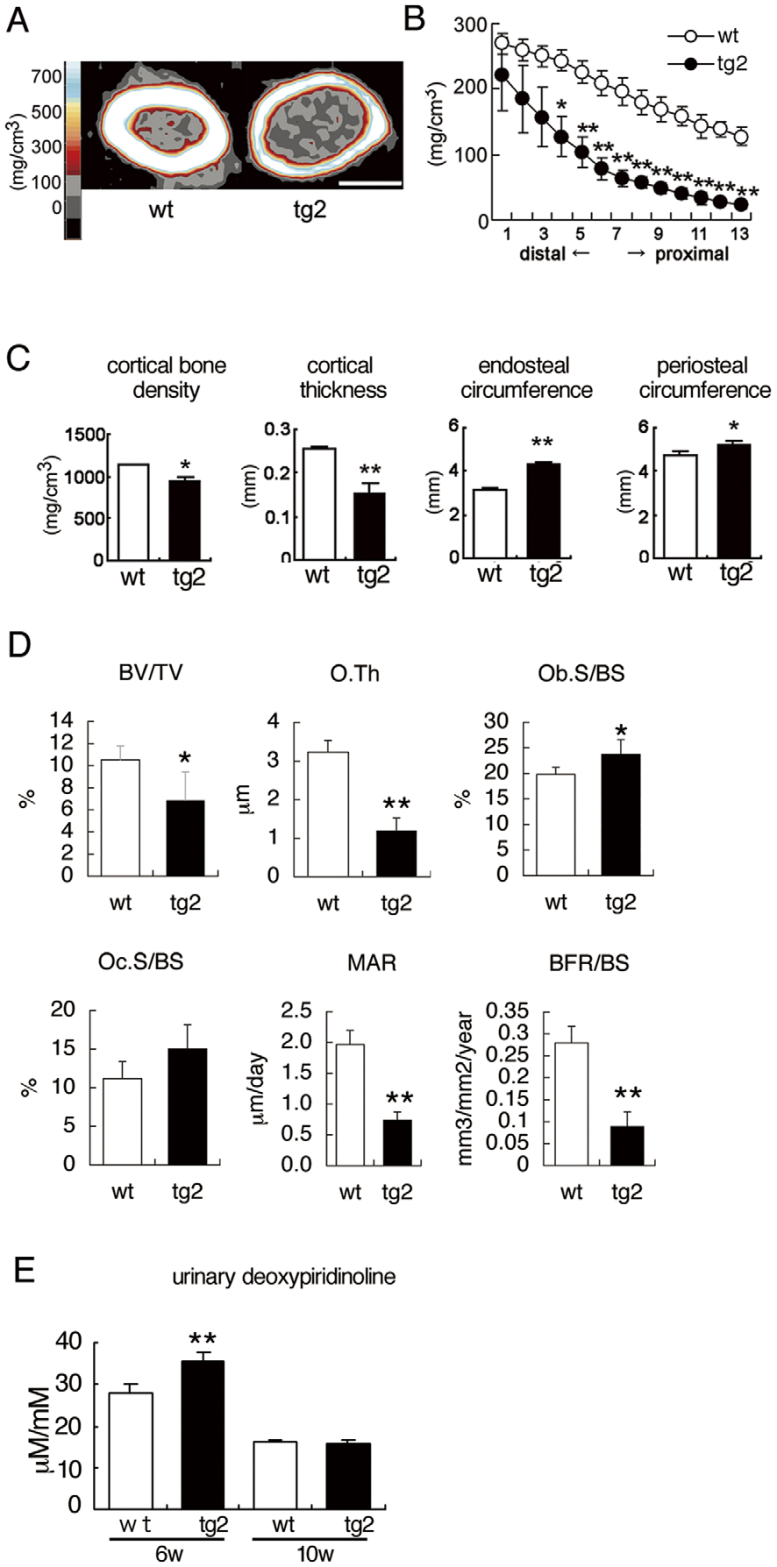
pQCT and bone histomorphometric analyses and bone resorption markers. (A–C) pQCT analyses of femurs from wild-type (wt) and tg2 mice at 10 weeks of age. A, pQCT images of the diaphyses of the femurs. Bar: 1 mm. B, The trabecular bone density in 13 equal cross-divisions of the metaphysial parts of the femurs was measured in wild-type (open circles) and tg2 (closed circles) mice. C, Cortical bone density, cortical thickness, endosteal circumference, and periosteal circumference of the diaphyses of the femurs were measured in wild-type (open columns) and tg2 (closed columns) mice. n = 5. (D) Bone histomorphometric analysis. Trabecular bone volume (bone volume/tissue volume, BV/TV), osteoid thickness (O.Th), osteoblast surface (Ob.S/BS), osteoclast surface (Oc.S/BS), mineral apposition rate (MAR), and bone formation rate (BFR/BS) were compared at 10 weeks of age. BS, bone surface. n = 5. (E) Urinary deoxypyridinoline levels at 6 and 10 weeks of age. n = 7–11. *P<0.05 and **P<0.01 vs. wild-type mice.

A pQCT analysis of 10-week-old tg2 mice revealed mineral density to be reduced in both the trabecular and cortical bones (Fig. 4A–C). pQCT and micro-CT analyses showed that the cortical bone was thin and both the endosteal and periosteal circumferences were enlarged (Fig. 4A, C and Fig. S3). A bone histomorphometric analysis revealed the trabecular bone volume (BV/TV) of tg2 mice to be two-thirds that of wild-type mice (Fig. 4D). Although the osteoblast surface (Ob.S/BS) in trabecular bone was increased in tg2 mice compared with wild-type mice, osteoblast function was impaired as shown by the severely reduced osteoid thickness (O.Th), mineral apposition rate (MAR), and bone formation rate (BFR/BS) (Fig. 4D). The osteoclast surface (Oc.S/BS) in tg2 mice was similar to that in wild-type mice, and the urinary deoxypyridinoline levels in tg2 mice were mildly increased at 6 weeks of age and similar to that in wild-type mice at 10 weeks of age (Fig. 4D, E). These findings suggest that the cortical bone in tg2 mice was immature, and the enlargement of the endosteal circumference was due to the immaturity of the cortical bone, which accelerates resorption, but not to enhanced osteoclastogenesis as previously described in *Runx2* and dominant-negative *Runx2* transgenic mice [9], [12], [13].

### Osteoblast differentiation at a late stage is inhibited in *Sp7* transgenic mice

**Figure 5:**
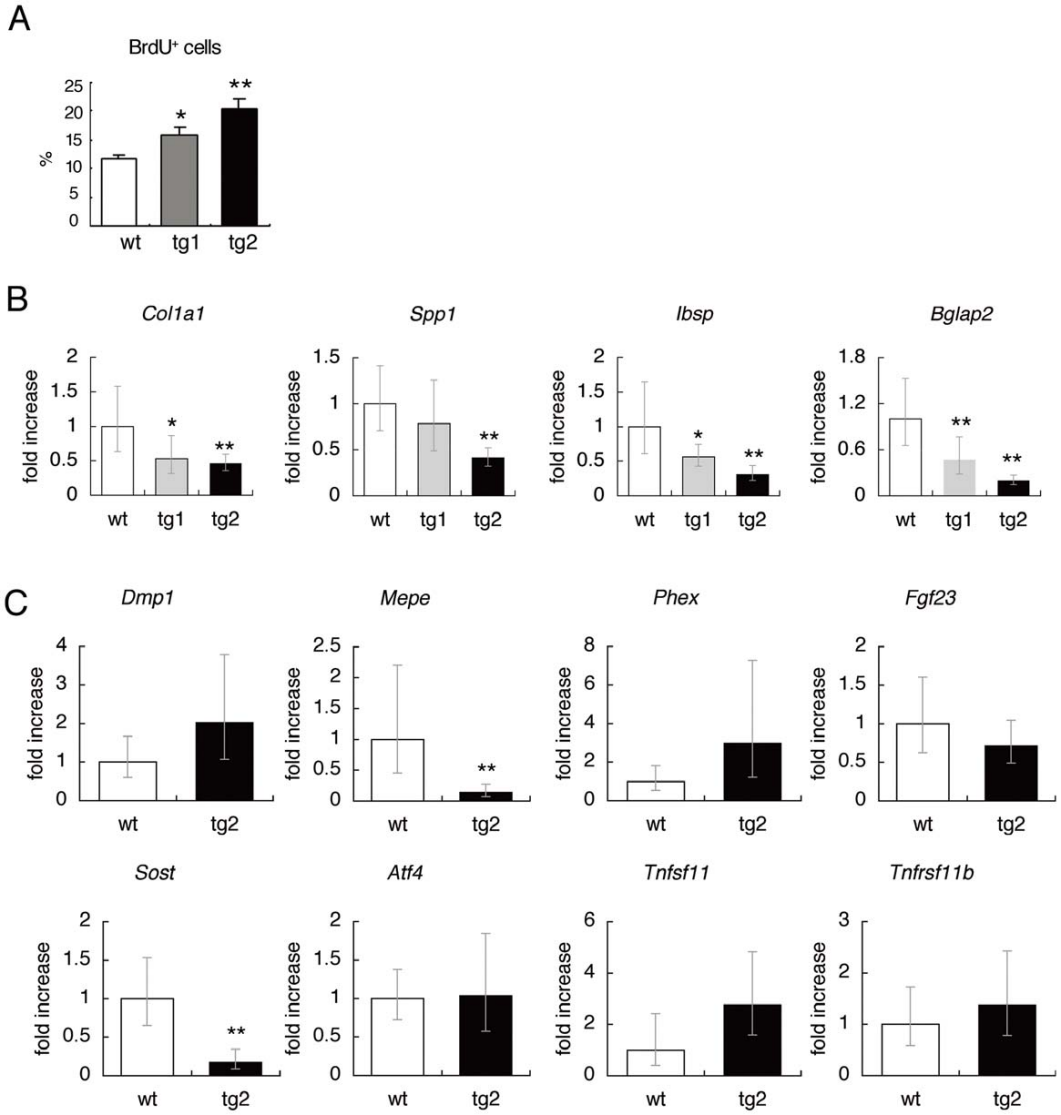
BrdU uptake and real-time RT-PCR analysis. (A) The percentage of osteoblasts positive for BrdU at 2 weeks of age. n = 4. (B, C) Real-time RT-PCR analysis. B, Expression of *Col1a1*, *Spp1*, *Ibsp*, and *Bglap2* was determined using RNA from whole bones of femurs and tibiae in wild-type (wt), tg1, and tg2 mice at 4 weeks of age. n = 5–8. *P<0.05 and **P<0.01 vs. wild-type mice. C, Expression of *Dmp1*, *Mepe*, *Phex*, *Fgf23*, *Sost*, *Atf4*, *Tnfsf11*, *Tnfrsf11b* was determined using RNA from the osteocyte fraction of femurs and tibiae in wild-type and tg2 mice at 12 weeks of age. n = 5. **P<0.01 vs. wild-type mice. The values of the wild-type mice were defined as 1, and relative levels are shown in B and C.

The osteoblast proliferation was enhanced in a manner dependent on the level of transgene expression (Fig. 5A). As osteoblast function was impaired in *Sp7* transgenic mice, we next examined the gene expression of major bone matrix proteins. A real-time RT-PCR analysis revealed the expression of *Col1a1*, *Spp1*, *Ibsp*, and *Bglap2*, to be significantly reduced in tg2 mice with the reduction in *Bglap2* expression being most severe (Fig. 5B). The expression of these genes was mildly or marginally reduced in tg1 mice. Combined with the histological, pQCT, and bone histomorphometric analyses (Fig. 3 and Fig. 4), these findings indicate that overexpression of *Sp7* promotes the proliferation but inhibits the differentiation at a late stage in osteoblasts.

As the osteocytes in *Sp7* transgenic mice showed morphological changes, we also examined the expression of the genes, which are highly expressed in osteocytes and/or mature osteoblasts, using osteocyte-enriched fraction (Fig. 5C). The expressions of *Mepe* and *Sost* were reduced in tg2 mice compared with wild-type mice. However, The expressions of *Dmp1*, *Phex*, *Fgf23*, *Atf4*, *Tnfsf11*, and *Tnfrsf11b* in tg2 mice were not significantly different from those in wild-type mice.

### SP7 and RUNX2 independently inhibit osteoblast differentiation at a late stage

**Figure 6:**
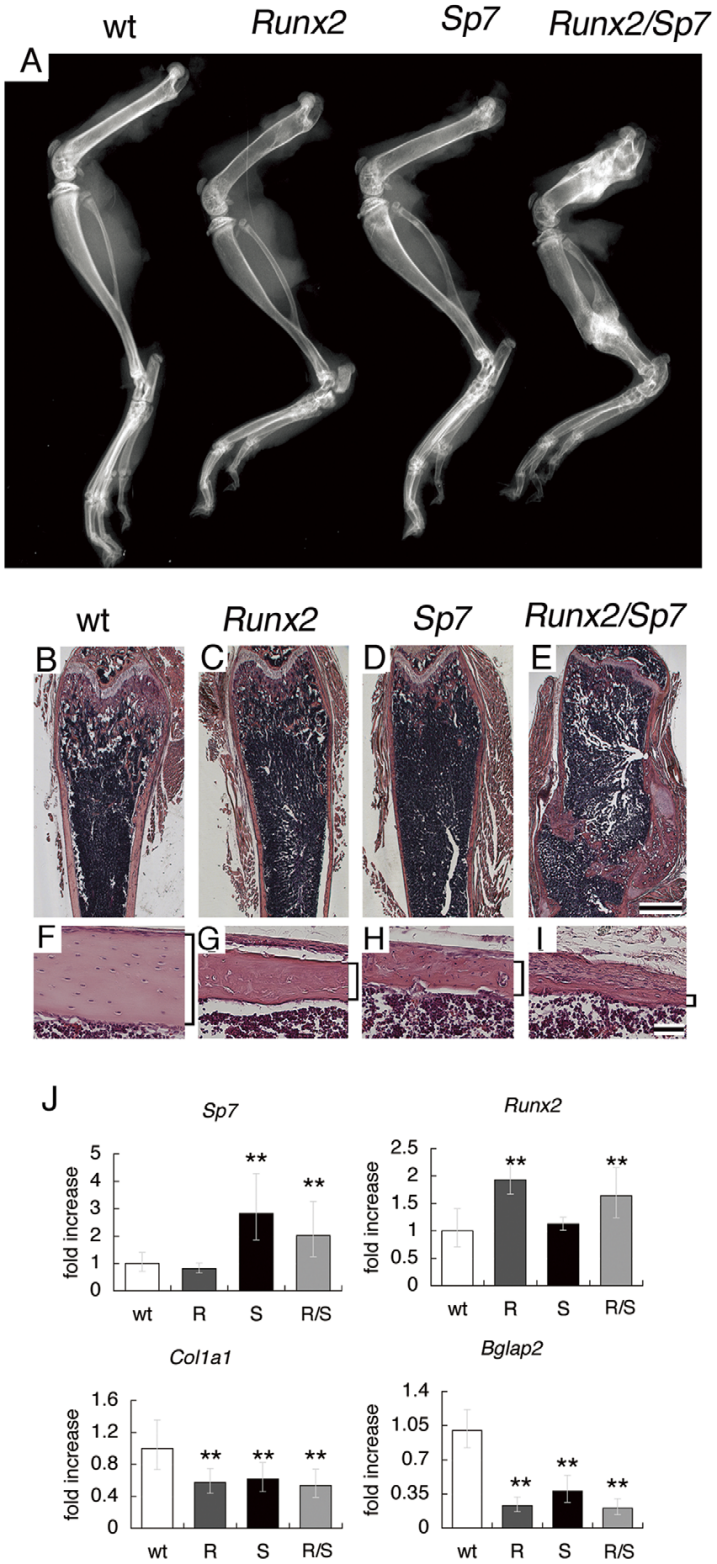
*Runx2/Sp7* double transgenic mice. (A) X-ray analysis of lower limb bones from a wild-type mouse (wt), *Runx2* transgenic mouse (*Runx2*), *Sp7* transgenic mouse (*Sp7*), and *Runx2/Sp7* double transgenic mouse (*Runx2/Sp7*) at 6 weeks of age. The double transgenic mouse (*Runx2/Sp7*) suffered from multiple fractures in the femurs, tibiae, fibulae, and calcanei, and the bone is most radiolucent in non-fracture regions. (B–I) H–E staining of sections from femurs of wild-type mice (B, F), *Runx2* transgenic mice (C, G), *Sp7* transgenic mice (D, H), and *Runx2*/*Sp7* double transgenic mice (E, I) at 6 weeks of age. Cortical bones are shown in F–I. The healing of fractures is observed in the double transgenic mouse (E). Cortical bones are shown by brackets. Bars: (B–E), 1 mm; (F–I) 50 μm. (J) Expression of *Sp7*, *Runx2*, *Col1a1*, and *Bgalp2* in calvaria of wild-type mice (wt), *Runx2* transgenic mice (R), *Sp7* transgenic mice (S), and *Runx2*/*Sp7* double transgenic mice (R/S) at 4 weeks of age was examined by real-time RT-PCR. For *Sp7* and *Runx2*, both endogenous expression and expression of the transgene were detected. **P<0.01 vs. wild-type mice. n = 4.

To further investigate the function of SP7 and RUNX2 in osteoblast differentiation at a later stage, we generated *Runx2*/*Sp7* double transgenic mice by mating tg2 mice with *Runx2* transgenic mice. The double transgenic mice showed severe osteopenia and suffered from more severe multiple fractures than tg2 or *Runx2* single transgenic mice (Fig. 6A–I). We compared the expression of *Sp7*, *Runx2*, *Col1a1*, and *Bglap2* among wild-type, *Runx2* transgenic, *Sp7* transgenic, and *Runx2*/*Sp7* double transgenic mice using RNA from calvaria, in which no fractures were seen in any of the groups (Fig. 6J). *Col1a1* expression was decreased and *Bglap2* expression was markedly decreased in *Runx2*, *Sp7*, and *Runx2*/*Sp7* double transgenic mice compared with wild-type mice. As *Sp7* expression was not upregulated in *Runx2* transgenic mice and *Runx2* expression was not upregulated in *Sp7* transgenic mice (Fig. 6J), these findings indicate that SP7 and RUNX2 independently inhibit osteoblast differentiation at a late stage.

### SP7 upregulates its own promoter

**Figure 7:**
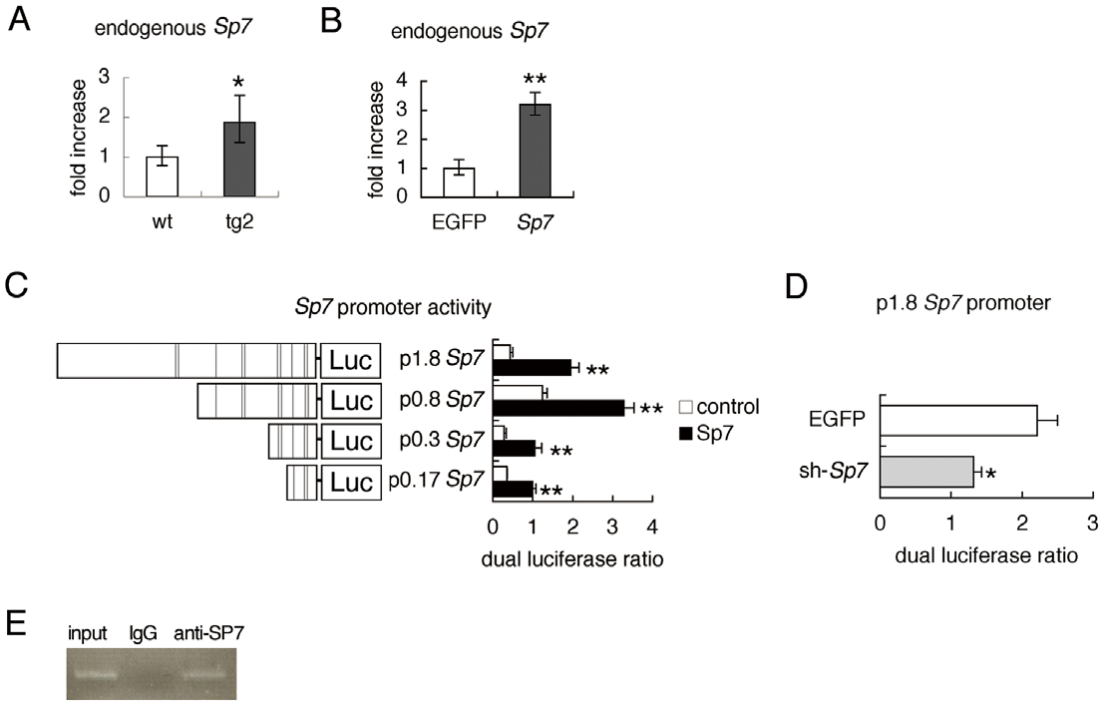
Positive regulation of *Sp7* promoter by SP7. (A, B) Real-time RT-PCR analysis. A, Endogenous *Sp7* expression in wild-type and tg2 mice at 18 weeks of age. RNA was prepared from tibiae and femurs, in which hematopoietic cells were flushed out. *P<0.05 vs. wild-type mice. n = 4–5. B, Osteoprogenitors isolated from wild-type calvaria using collagen gel were infected with adenovirus expressing EGFP or *Sp7* at 6×10^6^ pfu/well, and endogenous *Sp7* expression was examined. **p<0.001 vs. EGFP-expressing cells. The level in wild-type mice or EGFP-expressing cells was set at 1, and relative values are shown. (C, D) Reporter assays using the *Sp7* promoter. C, The reporter activity using the 1.8-kb *Sp7* promoter-luciferase (Luc) construct and the deletion constructs. Reporter assays were performed using 293T cells with (closed columns) or without (open columns) the *Sp7* expression vector. The lines in the schematic diagram of the *Sp7* promoter indicate putative SP1-binding sites. D, The reporter activity using the 1.8-kb *Sp7* promoter in ATDC5 cells infected with adenovirus expressing EGFP or sh-*Sp7* at 6×10^6^ pfu/well. *p<0.05 and **p<0.001 vs. control. (E) ChIP assay. DNA before immunoprecipitation (input) and after immunoprecipitation with anti-SP7 antibody or anti-IgG antibody was amplified by PCR using primers that amplify the region containing the proximal 170 bp of *Sp7* promoter.

The real-time RT-PCR analysis showed that the endogenous *Sp7* expression in tg2 mice was upregulated compared with that in wild-type mice (Fig. 7A). The endogenous *Sp7* expression was also increased but mildly in primary osteoblasts from *Sp7* transgenic mice in vitro (Fig. S4). Further, the adenoviral introduction of *Sp7* into wild-type osteoprogenitors induced endogenous *Sp7* expression (Fig. 7B). Thus, we examined *Sp7* promoter by reporter assay. The introduction of *Sp7* expression vector strongly activated the reporter vector containing the 1.8 kb *Sp7* promoter region (Fig. 7C). In the reporter assay using the serially deleted constructs, the proximal 170 bp was responsible for the activation (Fig. 7C). Further, sh-*Sp7* inhibited the activity of the reporter construct containing the 1.8 kb *Sp7* promoter region (Fig. 7D). ChIP assay showed that endogenous SP7 binds to the region containing the proximal 170 bp of *Sp7* promoter (Fig. 7E). These findings indicate that SP7 positively regulates its own promoter.

## Discussion

Introduction of sh-*Sp7* into primary osteoblasts inhibited osteoblast differentiation, showing that SP7 is required for osteoblast differentiation in vitro. However, overexpression of *Sp7* failed to induce *Alpl* expression and reduced *Bglap2* expression and mineralization at a late stage of osteoblast differentiation in vitro. The induction of osteoblast differentiation by SP7 is dependent on the cells and culture conditions in vitro [15], [16], [17], [18], [19], [20], [21], [22], [23], [24], [25], [26]. One possible explanation for these controversial results may be that SP7 plays an important role in osteoblast differentiation at an early stage but requires additional molecules to induce osteoblast differentiation. Further, SP7 may interact with different transcription factors at the early and late stages of osteoblast differentiation, and SP7 may interfere with the bindings or activities of the transcription factors, which are important for the late stage of osteoblast differentiation.

*Sp7* transgenic mice showed osteopenia due to the inhibition of osteoblast differentiation at a late stage, which was shown by woven bone structure in the cortical bone, reduced mineralization, reduced bone formation irrespective of the increased osteoblast density, and decreased expression of bone matrix protein genes especially *Bglap2*. Therefore, our experiments in vivo showed that SP7 inhibits osteoblast differentiation at a late stage and keeps the osteoblasts in an immature state. Although overexpression of *Runx2* also inhibits osteoblast differentiation at a late stage [9], [13], [27], SP7 and RUNX2 do so independent of RUNX2 and SP7, respectively, because the expression of *Sp7* and *Runx2* was not upregulated in *Runx2* transgenic mice and *Sp7* transgenic mice, respectively. However, RUNX2 induced *Sp7* expression in *Runx2* ^−/−^ calvarial cells, which retain the abilities to differentiate into chondrocytes and adipocytes [28]. Further, involvement of RUNX2 in *Sp7* expression was shown in C2C12 cells, C3H10T1/2 cells, and ATDC5 cells, which have characteristics of multipotent mesenchymal cells or chondroprogenitors [22], [29], [30]. Thus, RUNX2 seems to regulate *Sp7* expression at an early stage of osteoblast differentiation but not at a late stage. As RUNX2 and SP7 are essential for osteoblast differentiation at an early stage [5], [7], [31], our findings strengthen an idea that RUNX2 and SP7 play major roles in osteoblast differentiation at an early stage but not a late stage.

The deletion of *Sp7* in postnatal mice by CAG-CreER completely inhibits osteoblast differentiation and severely impairs bone formation, indicating that SP7 is essential for osteoblast differentiation at an early stage not only in embryos but also in postnatal mice [32]. Further, the deletion of *Sp7* using 2.3 kb *Col1a1* Cre results in an increase of trabecular bone but a decrease of cortical bone, and the deletion of *Sp7* in postnatal mice by 2.3 kb *Col1a1* CreERT results in a mild decrease of trabecular bone [6], [33]. *Bglap2* expression is reduced in both conditional *Sp7* knockout mice [6], [33], indicating that SP7 is still involved in osteoblast differentiation at the stage when preosteoblasts start to upregulate *Col1a1* expression and become immature osteoblasts. Combined with the data from the conditional *Sp7* knockout mice, our findings indicate that SP7 is required for postnatal bone development but osteoblast differentiation is inhibited at a late stage if the *Sp7* expression is maintained at a high level at the late stage of osteoblast differentiation.

Interestingly, the reduction in osteocyte processes was also observed in *Sp7*-deleted mice with CAG-CreER [32]. Thus, either overexpression or deletion of *Sp7* reduced osteocyte processes. In *Sp7* transgenic mice, the number of osteoblast processes was also reduced. Therefore, SP7 may be involved in the regulation of process formation of osteoblasts and osteocytes. It is also possible that disturbance of the differentiation of osteoblasts at a late stage is responsible for the reduction in the number of processes in osteoblasts and osteocytes, because the number of processes was also reduced in osteoblasts and osteocytes in *Runx2* transgenic mice [9] (data not shown). The reduction of *Mepe* expression and *Sost* expression in the osteocytes of *Sp7* transgenic mice may reflect the impaired mineralization and immaturity of the osteocytes, respectively [34], [35], [36].

Osteoblast proliferation was enhanced in *Sp7* transgenic mice. The enhanced osteoblast proliferation by SP7 was also observed in NIH3T3 fibroblasts and bone marrow stromal cells in vitro [16], [18]. Thus, SP7 seems to have an ability to induce osteoblast proliferation. It is also possible that the enhanced osteoblast proliferation was due to the inhibition of osteoblast differentiation at a late stage, which results in the accumulation of immature osteoblasts that retain the ability to proliferate.

In conclusion, the sustained *Sp7* expression at high levels during osteoblast differentiation inhibited osteoblast differentiation at a late stage. Thus, the stage-dependent regulation of *Sp7* mRNA expression and protein activity during osteoblast differentiation is essential for bone formation. The auto-regulatory loop of SP7 may play an important role in maintaining a sufficient level of SP7 at an early stage of osteoblast differentiation.

## Supporting Information

Figure S1 (A) Quantification of formazan's incorporation into primary osteoblasts. The cells were infected with adenovirus (6×10^6^ pfu/well) carrying EGFP, *Sp7* or sh-*Sp7* at confluence, and MTT assay was performed after 4 days of culture in osteogenic media. Data is presented as mean ± S.D. of absorbance values measured on 4 wells. (B–E) SP7 inhibits mineralization. Primary osteoblast cultures infected with retrovirus carrying EGFP (B, D) or *Sp7-*EGFP (C, E). B and C, Dark field images showing cells expressing the respective transgene. Von Kossa staining (D, E) was performed 10 days after infection.
(TIF)

Figure S2 Histological analysis. H–E staining of sections of tibiae from wild-type (A, B, E, F, I, J, M, N) and tg2 (C, D, G, H, K, L, O, P) mice at 1 week (A–D), 2 weeks (E–H), 4 weeks (I–L) and 10 months (M–P) of age. Boxed regions in A, C, E, G, I, K, M, O, are magnified in B, D, F, H, J, L, N, P, respectively. Scale bars: (A,C,E,G,I,K,M,O) 200 μm, (B,D,F,H,J,L,N,P) 50 μm.
(TIF)

Figure S3 Micro-CT analysis. (A–B) Micro-CT images of cortical bones in femora of wild-type (A) and tg2 (B) mice at 15 weeks of age. (C) Cortical bone ratio (bone volume/total volume). (D) Medullary ratio (medullary volume/total volume). (E) Endosteal circumference. (F) Periosteal circumference. Parameters were measured on cortical bone of the femoral diaphysis. *P<0.05, **P<0.005 vs. wild-type mice. n = 3.
(TIF)

Figure S4 Real time RT-PCR analysis. (A) Exogenous and endogenous *Sp7* expression. (B) Endogenous *Sp7* expression. Primary osteoblasts from wild-type and *Sp7* transgenic mice were plated on 24-well plates at a density of 3×10^5^ cells/well and RNA was extracted 2 days later. n = 6 .**P<0.01 vs. wild-type primary osteoblasts.
(TIF)

Procedures S1 MTT assay.
(DOC)

Procedures S2 Retrovirus infection.
(DOC)
