# Supplementary figures and images for: SP7 Inhibits Osteoblast Differentiation at a Late Stage in Mice
Source: PLoS One. 2012 Mar 2;7(3):e32364. doi: 10.1371/journal.pone.0032364 (PMC3292551; doi:10.1371/journal.pone.0032364)

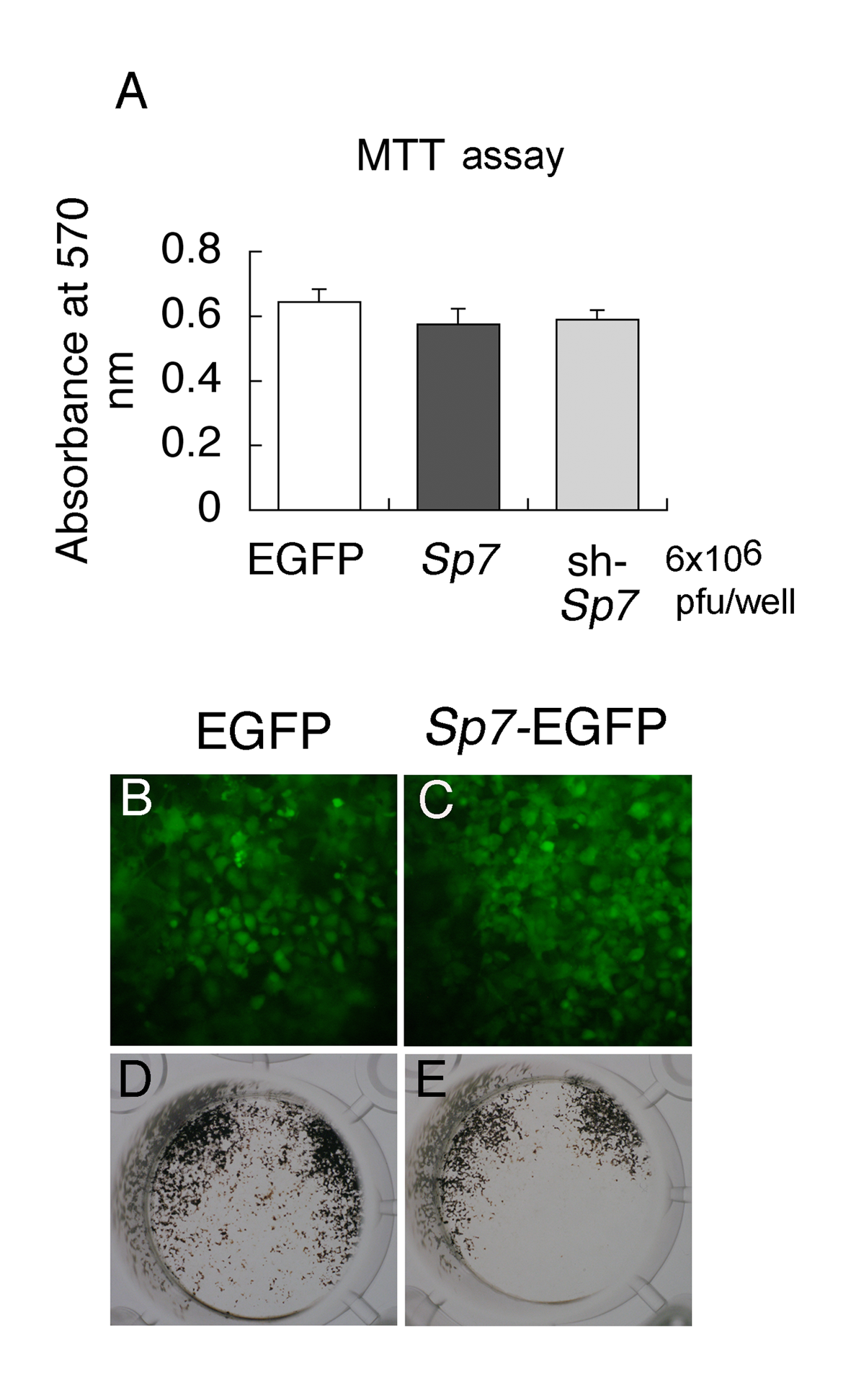

Supplement: Figure S1 — (A) Quantification of formazan's incorporation into primary osteoblasts. The cells were infected with adenovirus (6×10^6^ pfu/well) carrying EGFP, Sp7 or sh-Sp7 at confluence, and MTT assay was performed after 4 days of culture in osteogenic media. Data is presented as mean ± S.D. of absorbance values measured on 4 wells. (B–E) SP7 inhibits mineralization. Primary osteoblast cultures infected with retrovirus carrying EGFP (B, D) or Sp7-EGFP (C, E). B and C, Dark field images showing cells expressing the respective transgene. Von Kossa staining (D, E) was performed 10 days after infection. (TIF) [file pone.0032364.s001.tif]

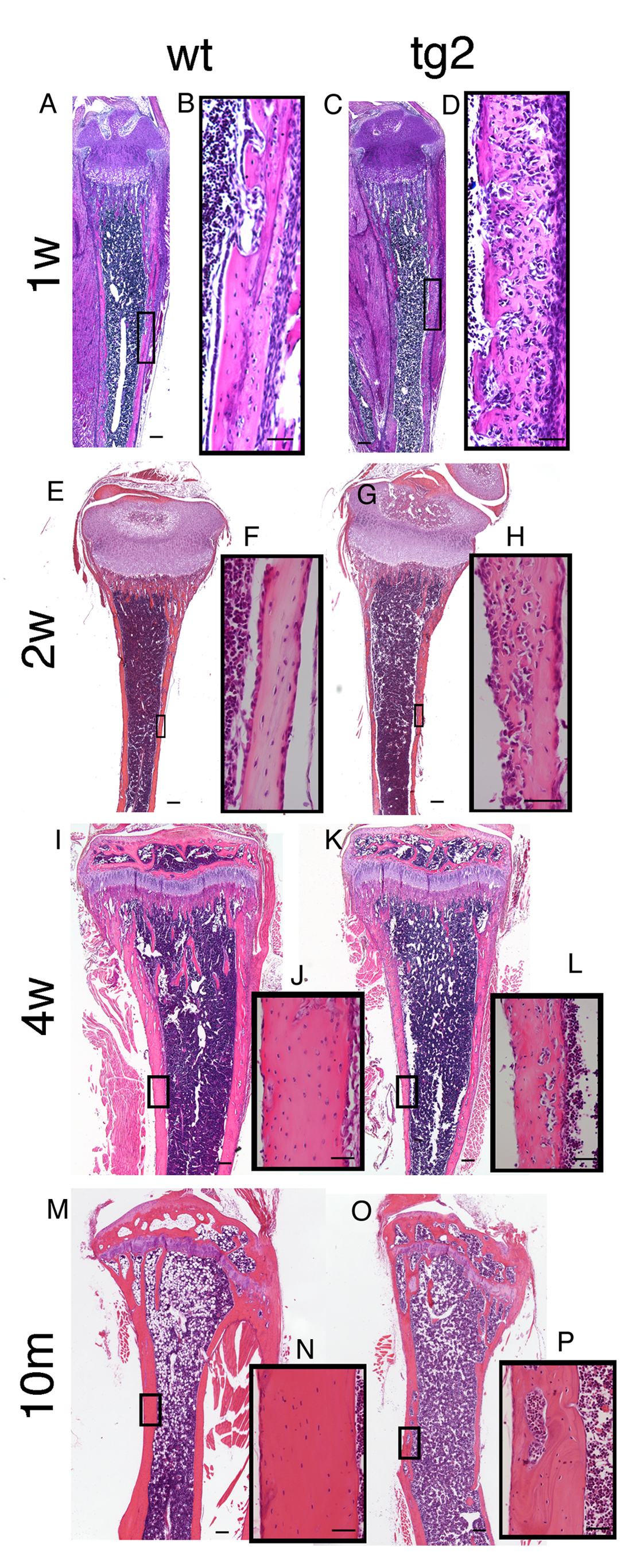

Supplement: Figure S2 — Histological analysis. H–E staining of sections of tibiae from wild-type (A, B, E, F, I, J, M, N) and tg2 (C, D, G, H, K, L, O, P) mice at 1 week (A–D), 2 weeks (E–H), 4 weeks (I–L) and 10 months (M–P) of age. Boxed regions in A, C, E, G, I, K, M, O, are magnified in B, D, F, H, J, L, N, P, respectively. Scale bars: (A,C,E,G,I,K,M,O) 200 μm, (B,D,F,H,J,L,N,P) 50 μm. (TIF) [file pone.0032364.s002.tif]

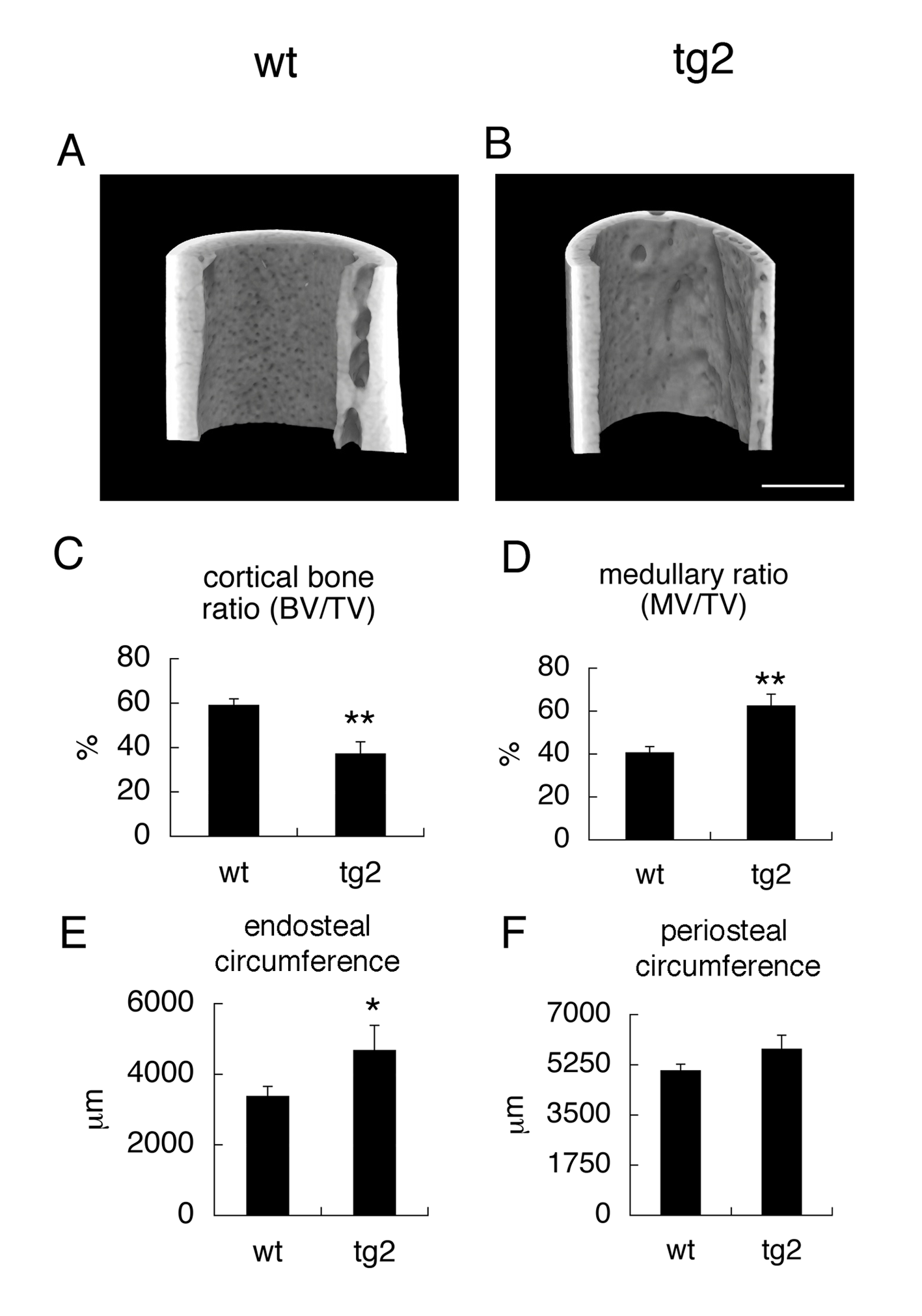

Supplement: Figure S3 — Micro-CT analysis. (A–B) Micro-CT images of cortical bones in femora of wild-type (A) and tg2 (B) mice at 15 weeks of age. (C) Cortical bone ratio (bone volume/total volume). (D) Medullary ratio (medullary volume/total volume). (E) Endosteal circumference. (F) Periosteal circumference. Parameters were measured on cortical bone of the femoral diaphysis. *P<0.05, **P<0.005 vs. wild-type mice. n = 3. (TIF) [file pone.0032364.s003.tif]

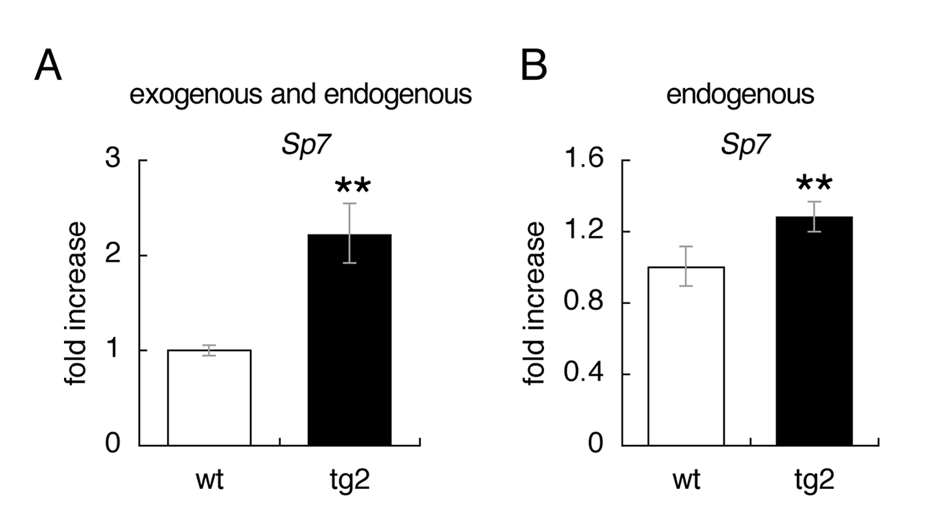

Supplement: Figure S4 — Real time RT-PCR analysis. (A) Exogenous and endogenous Sp7 expression. (B) Endogenous Sp7 expression. Primary osteoblasts from wild-type and Sp7 transgenic mice were plated on 24-well plates at a density of 3×10^5^ cells/well and RNA was extracted 2 days later. n = 6 .**P<0.01 vs. wild-type primary osteoblasts. (TIF) [file pone.0032364.s004.tif]
