# Supplementary material for: SP7 Inhibits Osteoblast Differentiation at a Late Stage in Mice
Source: PLoS One. 2012 Mar 2;7(3):e32364. doi: 10.1371/journal.pone.0032364 (PMC3292551; doi:10.1371/journal.pone.0032364)
Supplement: Procedures S1 — MTT assay. (DOC) [file pone.0032364.s005.doc]

Procedures S1. MTT assay.

Primary osteoblasts were infected with adenovirus expressing EGFP, *Sp7* or sh-*Sp7***.** After 4 days of culture, the osteogenic differentiation media was changed to phenol red free media supplemented with 0.5mg/ml 3-[4,5-dimethylthiazol-2-yl][^4^](#_ENREF_4)-2,5-diphenyl tetrazolium bromide (MTT). After incubation for 30 minutes, the precipitated blue formazan was released from the cells using dimethyl sulfoxide (DMSO), and the colorimetric evaluation was performed using a spectrophotometer at a wavelength of 570 nm.
