# Supplementary material for: SP7 Inhibits Osteoblast Differentiation at a Late Stage in Mice
Source: PLoS One. 2012 Mar 2;7(3):e32364. doi: 10.1371/journal.pone.0032364 (PMC3292551; doi:10.1371/journal.pone.0032364)
Supplement: Procedures S2 — Retrovirus infection. (DOC) [file pone.0032364.s006.doc]

Procedures S2. Retrovirus infection.

pMX bicistronic vectors expressing *Sp7* and EGFP or EGFP alone were transfected into a packaging cell line Plat-E. Forty-eight hours later, medium containing the retrovirus was harvested, supplemented with 8μg/ml polybrene, and used to infect pre-confluent primary osteoblasts.
